# Foal sex in Thoroughbred horses: related factors

**DOI:** 10.1590/1984-3143-AR2023-0073

**Published:** 2024-08-05

**Authors:** Jonas Gomes Flores, Verônica La Cruz Bueno, Henrique Boll de Araujo Bastos, Sandra Mara da Encarnação Fiala Rechsteiner

**Affiliations:** 1 Histologia e Reprodução Equina (HISTOREP), Faculdade de Veterinária, Universidade Federal de Pelotas, Pelotas, RS, Brasil; 2 Laboratório de Reprodução Animal (REPROLAB), Faculdade de Veterinária, Universidade Federal do Rio Grande do Sul, Porto Alegre, RS, Brasil

**Keywords:** concept, equine, influence

## Abstract

Reproductive biotechniques in the equine species have advanced in the last decade and horse breeders have started to question the possibilities of interfering in the determination of foal sex. The aim of the present study was to verify whether the variables mares and stallion’s age, side of the ovary containing the preovulatory follicle, preovulatory follicle diameter, time between breeding and ovulation, and ovulation inducing hormones influence the sex of the foal. A total of 259 reproductive cycles of 160 mares and 22 Thoroughbred stallions were used. Statistical analysis was performed using R software, including Pearson's chi-square test and logistic regression. Of the total foals born, 136 were males (52.51%) and 123 were females (47.49%). In mares that ovulated with -24h after ovulation induction, 104 foals (54.74%) were males and 86 (45.26%) were females, while in mares that ovulated with +24h, 32 foals (46.38%) were males and 37 (53.62%) were females. Stallions up to 15 years old had 44.14% (n=49) females and those over 15 years had 49.66% (n=73) females. The simple logistic regression model showed that mares and stallions under 15 years old, mares with ovulation time less than 24 hours and treated with Deslorelin had a higher probability of having male foals, but the Pearson's chi-square test showed that foals gender were not influenced by the variables studied.

## Introduction 

The problem of the evolution of the sex ratio is studied since the time of Fisher (Smith 1980; Edwards 2000). Some theories have been proposed to explain the sex ratio in animals: the Trivers–Willard hypothesis (Trivers and Willard 1973), local resource competition (Silk 1983) and the developmental asynchrony hypothesis for sex ratio manipulation (Krackow 1995), but none has been confirmed (Cameron 2004) or can be used for horses (Cameron et al., 1999, Cameron 2004). In mammals, oocytes produced by females always contribute the same sex ‘X’ chromosome, but those produced by males contribute either an ‘X’ or a ‘Y’ sex chromosome, which dictates offspring sex at conception. Since the number of gametes that carry both the ‘X’ and the ‘Y’ chromosome during spermatogenesis is equal, the probability that an embryo is a male or a female is 50%. In humans and mices the offspring sex ratio can differ significantly from 1:1 (Abecia et al., 2016; Gutiérrez-Adán et al., 2000).

For horse breeders, the interest in breeding a foal with a predetermined sex is subjective, some of the possible reasons: replacing a mare; generate a potential stallion of noble lineage (Samper et al., 2012). In Thoroughbred, reproductive biotechnologies, such as artificial insemination and embryo transfer, are prohibited by the association, which does not allow laboratory control to choose the foal's sex.

In humans, the study of the influence factors related to the sex of the baby has been an object of study for many years (Wilcox et al. 1995). The desire of the human species to control the gender of its progeny prior to conception has always existed. Reasons range from family balancing to culturally imposed preference for boys to prevention of sex-linked hereditary diseases (Noorlander et al., 2010). In animals, controlling sex ratio permits faster genetic progress, higher productivity, improves animal welfare and helps to reduce environmental impact due to the elimination of the unwanted sex (Rath et al., 2009). An important feature is must be fact the gestation length of horses is longer than most other domestic animals. When coupled with an extended gap from birth to the onset of training, several years, and hence several foal crops, are needed to determine the worthiness of a sire or dam as producers of quality offspring (Scoggin et al. 2015).

Several studies in humans, horses, cattle, sheep concluded that certain factors such as: natural mating, body condition of the mother, how pregnant and the age of the parents would have an influence on the sex of the product (Martinez et al., 2004; Gutiérrez-Adán et al., 1999; Cameron and Linklater 2007; Giraldo et al., 2010; Hylan et al., 2009; Santos et al., 2015). The relations of an offspring to its parents are complex, and the ways in which a parent may influence the characteristics of its offspring are many (Liu et al., 2011). In the equine species, sex determination of the conceptus is of growing interest for the breeding industry (Aurich and Schneider 2014).

The sex ratio is an important topic in equine reproduction (Cameron et al., 1999; Santos et al., 2015; Hall et al., 2022). The aim of the present study was to verify whether the variables mares and stallion’s age, side of the ovary containing the preovulatory follicle, preovulatory follicle diameter, time between breeding and ovulation, and ovulation inducing hormones influence the sex of the foal.

## Methods

### Animals

The study was not submitted to the Animal Ethics Committee because the researchers did not have contact or handled the animals.

The present retrospective study evaluated data during the breeding seasons from 2011 to 2015, the animals belonged to different breeding centers of the Bagé/RS and Aceguá/RS – Brazil (31°24'06.1''S 54°07'47.5''W) and (31°30'16.0''S 54°07'45.5''W).

Data were recorded of 259 cycles of 160 mares, natural mating by 22 thoroughbred stallions, mares weight ranged between 450 and 600 Kg and stallions weight ranged between 500 and 650kg. The mares were natural mating with stallions chosen by the owners.

Mares were kept into small groups of approximately 15 animals, in natural pastures, in areas ranging from 10 to 15 hectare, fed oat grains BID with free access to water.

The stallions were kept during the day in small individual paddocks of approximately 100 m^2^ and at night they were housed in 5x5m stables. They were fed 8kg of oat grains, 1kg commercial feed and alfalfa hay daily, with free access to water.

### Reproductive management

The reproductive management of the animals was carried out by the veterinarians responsible for the breeding centers. Mares were examined for follicular control by transrectal palpation and ultrasound of the genital tract (Aloka, SSD-500), with linear transducer 5,0 MHz.

Mares were monitored every 24h or 48h depending on the condition of the mare. Once mares were in estrus and a follicle >35 mm in diameter, marked uterine edema and receptivity to the stallion were evidenced, the ovulation was induced with Human Chorionic Gonadotropin (Chorulon®, Intervet), 2.500UI IV (n= 72) or deslorelin acetate (Sincrorrelin®, OuroFino) 3 ml/IM equivalent to 750ug (n= 187), 69 mares were induced to ovulate at the time of natural mating and 190 mares were induced 24h before natural mating. The mares were monitored every 24 h from the natural mating until ovulation and only those that were natural mating only once per cycle were part of the study.

Prior to natural mating, the mare’s tail was bandaged and tied away from contact with the vulva and perineum. The vulva was scrubbed clean and rinsed thoroughly with water. The vulva and perineal area were dried.

### Experimental design

The studied variables on the sex of the foal were: mares and stallion’s age, side of the ovary containing the preovulatory follicle, preovulatory follicle diameter, time of the breeding in relation to time of ovulation, and ovulation inducing hormones. Mares were distributed into three groups according to age: up to 8 years old (n= 123), 9 to 14 years old (n=110) and over 14 years old (n=26). The stallions were distributed into two groups: up to 14 years old (n=11) and 15 years old or more (n=11).

During the exams, the studied variables were registered: ovary that had the preovulatory follicle, diameter of the preovulatory follicle, ovulation inducing hormones used for ovulation induction (hCG or Deslorelin), age of the mare and the stallion, the moment when ovulations occurred from the moment of natural mating (up to 24h or more than 24h after natural mating). The diagnosis of pregnancy was made at 14 days.

### Statistical analysis

Data were analyzed using computer software R, 3.4.1. version. For the univariate descriptive analysis of the data, averages were calculated, standard deviation and minimum, median and maximum values for continuous variables and the frequencies of each category for categorical variables. Pearson's chi-square test was used to analyze the influencing factors and to verify the association of each factor with the sex of the foals, differences P < 0.05 were considered significant. Logistic Regression was used to calculate the Odds Ratio and test the influence of factors on the foal's sex.

The distribution used for the study of the research variables was Binominal. The logistic regression model was adjusted according to Hosmer et al., (2000), using the criterion of maintaining only variables with a p-value greater than 0.25 in the model and choosing the best model using the Akaique criterion (AIC).

Continuous variables were studied: age of mare and age of stallion, despite the variables age of the mare and age of the stallion being of a continuous nature, they were categorized for analysis, divided into categories up to 8 years, 9 to 14 years old and over 14 years old to mares, and up to 15 years old and 15 years old or more to stallions and categorical variables: ovary; ovulation induction; ovulation time; and sex of foals.

### Age of mares

$$\text{P (foal sex = male)=}\frac{1}{1+e^{-0(\beta o+\beta 1x)}}$$ (1)

In the equation, X is an indicator binary variable that takes on value 1, in the event that the mare's age is up to 8 years; from 9 to 14 years old or older than 14 years old and 0 otherwise.

### Age of stallions

$$\text{P (foal sex = male)=}\frac{1}{1+e-0\left(\beta 0+\beta 1X\right)}$$ (2)

In the equation, X is an indicator binary variable that takes on value 1, in the case where the stallion's age is less than or equal to 15 years and 0 otherwise.

### Ovary

$$\text{P (foal sex = male)=}\frac{1}{1+e-0(\beta 0+\beta 1X)}$$ (3)

In the equation, X is an indicator binary variable that takes on value 1, in the case that the ovary was the Right and 0 otherwise.

### Ovulation inductor

P (foal sex = male)=11+e_0(β0+β1X) (4 )

In the equation, X is an indicator binary variable that takes on value 1, in the case that the inductor used was deslorelin acetate and 0 otherwise.

### Ovulation time

$$\text{P (foal sex = male)=}\frac{1}{1+e\_0(\beta 0+\beta 1x)}$$ (5)

In the equation, X is an indicator binary variable that takes on value 1, in the event that the ovulation time is less than or equal to 24 hours and 0 otherwise.

## Results

### Univariate descriptive analysis

The stallions’s mean age was 9.2 (± SD 3,37) years, with a minimum age of 2 and a maximum of 20 years and in the stallions 13.7 (± SD 5,99) years, 4 years (minimum) and 23 years (maximum), respectively (Table 1). The average size of the preovulatory follicle was 44.2mm (± SD 4,14), with a minimum of 35mm and a maximum of 56mm. In the sample collected, in 123 of the births of foals, equivalent to 47.4% of the total, the age of the mare at the time of mating was less than eight years. In 110 of the foal births, equivalent to 42.4% of the total, the age of the mare at the time of mating was between 9 and 14 years. Only 26 births, equivalent to 10%, were observed in mares aged over 14 years. Among the stallions, 148 (57.14%) of the foals born were from horses that were over fifteen years old at the time of mating and 111 (42.86%) of the foals were from horses that were less than 15 years old at the time of mating.

**Table 1:** Result from retrospective study with evaluation of factors influencing the sex of foals.

	**Female**	**Male**
Time of ovulation - 24h	86 (45.26%)	104 (54.74%)
Time of ovulation + 24h	37 (53.62%)	32 (46.38%)
Up to 8 years old (mares)	57 (46.34%)	66 (53.66%)
9 - 14 years old (mares)	52 (47.27%)	58 (52.73%)
Over 14 years old (mares)	14 (53.85%)	12 (46.15%)
Up to 15 years old (stallions)	49 (44.14%)	62 (55.86%)
Over 15 years old (stallions)	73 (49.66%)	74 (50.34%)
Deslorelin acetate	84 (44.92%)	103 (54.08%)
hCG	39 (54.17%)	33 (45.83%)
Right ovary	57 (46.72%)	65 (53.28%)
Left ovary	66 (48.18%)	71 (51.82%)

In 137 (52.9%) pregnancies, ovulations occurred in the left ovary (Table 1), while 54.3% (n = 82) of pregnancies occurred in the right horn. In 72.2% (n = 187) of pregnancies, the ovulation inductor used was Deslorelin and in 27.8% (n = 72) hCG. The percentage of born foals, 52.51% (n = 126) were male, while 47.49% (n = 123) were female.

### Factors influencing the foal's sex

By Pearson's chi-square test, there was no sample evidence of an association between the time of ovulation in relation to the time of natural mating with the sex of the foals (p-value = 0.2936), there was no association between the mare’s age at the time of natural mating and the sex of the foals (p-value = 0.7833), there was no association between the stallion’s age at the time of natural mating and the sex of the foals (p-value = 0.4516).

There was no association between the used ovulation inductor and the sex of the foals (p = 0.2316), between the ovary in which ovulation occurred and the sex of the foals (p = 0.913) as seen in Table 1.

### Simple logistic regression

According to the simple logistic regression model, the probability of mares under the age of 15, at the time of natural mating is 41, 59% higher birth of male foals than those over 15 years old. Similar results as observed among stallions however the probability of male foals being born was 24,82% higher between those with up to 15 years old than stallions with over 15 years old.

Our study showed that mares with time of ovulation less than 24 h, have a higher probability of birth male foals (39,83%). The results of the Delosrelin showed higher probability 44,91% of birth male foals than hCG. In the right ovary, the probability of birth male foal was 6,00% higher than in the left ovary.

No odds ratio was statistically significant. The biggest consequence of this is not being able to accurately extend these results to the entire target population, however they are valid for the sample (The p-values are shown in Table 2 together with the Confidence Intervals for the Odds Ratios).

**Table 2:** Simple logistic regression models of the variables that influencing the sex of foals . *IC – confidence interval

	**Odds ratio**	IC-5%*	IC-95%*	**p-valor**
Mares up to 15 years old	1.4159	0.5403	3.8266	0.4793
Stallions up to 15 years old	1.2482	0.7616	2.0519	0.3799
Time of ovulation up to 24h	1.3983	0.8053	2.4394	0.2345
Deslorelin	1.4491	0.8406	2.5111	0.1828
Time of pregnancy – over 335d	2.1600	0.9727	5.0543	0.0641
Right ovary	1.0600	0.6501	1.7298	0.8151

## Discussion

Our study performed Pearson's chi-square test to analyze the influencing factors and to verify the association of each factor with the sex of the foals and showed that the foals gender were not influenced by the variables studied. However the Logistic Regression was possible to calculate the influence of factors on the foal's sex. Studies demonstrate that the age of parents can influence the descendants sex. (Saltz and Rubenste 1995 ; Nicolich et al., 2000; Santos et al., 2015).

While Pearson's chi-square test suggested that the sex of the foals was not influenced by the variables studied, Logistic Regression analysis showed probability. We believe that the results of Logistic Regression complement, rather than contradict, those of the chi-square test. While the chi-square test assesses the association between categorical variables, such as the sex of the foals and other variables, Logistic Regression goes further by calculating the probability of male birth based on the variables studied. Thus, both analyses are important as they provide different and complementary perspectives on the influence of factors on the sex of the foals. While the chi-square test tells us if there is an association between variables, Logistic Regression helps us better understand how these variables influence the sex of the foals. Therefore, we consider that both analyses together offer a more complete and robust view of the studied phenomenon (Hosmer et al., 2000).

Our research showed that the probability of male birth fraction, declines with increasing age of mare and stallion. These results agree with studies in humans that showed male birth fraction declines with increasing age of either parent (Nicolich et al., 2000), and humans sex ratio decreased with increasing number of children per plural birth and with increasing paternal. Current knowledge suggests that parental age has many influences on the offspring in humans (Liu et al., 2011).

In relation to the age of mares, we showed that the probability of mares under the age of 15 at the time of natural mating is 41,59% higher birth of male foals than those over 15 years old. The first study that evaluated the influence of parental age on the sex ratios in horses, concluded that the aging of the mares influenced the sex ratios of their offspring by increasing the probability of female offspring (Santos et al., 2015). This result corroborates with our findings where it was verified that the age of the mares had influence on the sex of the offspring.

Mare's results were similar observed in the stallions older than 15 years. The probability of male foals born was 24,82% higher between those with up to 15 years old than stallions with over 15 years old. In agreement with other studies that showed, a negative correlation was found between the age and the offspring sex ratio until 19 years of age in stallions (Santos et al., 2015). Sartorelli et al., (2001) observed a similar frequency of the Y- and X-bearing spermatozoa (47.4% vs. 52.6%) in the ejaculates of 59- to 74-year-old men and a significantly higher frequency of Y-bearing than that of X-bearing spermatozoa (55.5%) in the ejaculates of 23- to 29-year-old men. Similarly, Stone et al. (2013) observed a negative correlation between the age and the proportion of Y-bearing spermatozoa in the semen of men older than 40 years. The male effect on the sex ratio might be related to the selective output of the X- or Y-bearing spermatozoa. Variations in the Y:X–bearing spermatozoa ratio of various species may be caused by several factors, which include exposure to environmental contaminants, fertility, sexual rest, male attractiveness, diet, and age (Santos et al., 2015). This result corroborates with our findings where it was verified that the age of the mares had influence on the sex of the offspring.

However, by Pearson's chi-square test there was no association between the age of the stallion at the time of natural mating and the sex of the foals. In studies with wild equids, the parental age was not a factor that affected the offspring sex ratio (Aurich and Schneider 2014; Cameron et al., 1999).

According to the simple logistic regression model, mares with time of ovulation less than 24h after ovulation induction, have a higher probability of birth male foals (39,83%). Many studies support our results. Hall et al., (2022) conducted a study with objective was to determine the effect of period between breeding and ovulation (PBBO) on the sex of the foal results indicated that the closer a mare is natural mating to ovulation with fresh semen, it is more likely that she will have a colt. Harlap (1979) shows that the timing of coitus in respect to ovulation seems to influence the sex ratio. Researchers (Martinez et al., 2004; Gutiérrez-Adán et al., 1999; Zarutskie et al., 1989) studying the interference of insemination time or natural mating in relation to the time of ovulation found differences in the percentage of sex calves, lambs and babies born respectively, which demonstrate that when insemination or natural mating is carried out close to the time of ovulation a greater number of males is generated. In ewes differences in the sex ratio were obtained when the authors compared the sex of the offspring of ewes inseminated during the 5h preceding ovulation (more females) with those inseminated during the 5h after ovulation (more males) (Gutiérrez-Adán et al., 1999). Other study in cattle observed that, delaying the AI significantly increases the percentage of males (Martinez et al., 2004). Corroborating our findings, a study sought to determine the effect of the period between breeding and ovulation on foal sex. Foaling records (n = 288) from the breeding seasons of 2015 to 2018 from 2 veterinary hospitals were used. The results indicated that the closer a mare is bred to ovulation, the more likely she is to have a foal (Hall et al., 2022).

The results of the Deslorelin showed higher probability 44,91% of birth male foals than hCG. Corroborating our findings, two hundred fifty-six children were born in 195 births from 176 women that conceived after human menopausal gonadotropin/human chorionic gonadotropin (hMG/hCGJ-induced ovulation, the secondary sex ratio was 50% male to 50% female births. The same trend was observed for single births and for twins (Ben-Rafael et al., 1986). The sex ratio of the fetuses from mice treated with pregnant mare’s serum (PMS)/human chorionic gonadotropin (HCG) to induce ovulation did not differ appreciably from that of spontaneously ovulated controls (Sakai and Endo 1987).

The results of the Deslorelin showed higher probability 44.91% of birth male foals than hCG. The administration of hormones to mares during breeding management is an essential tool for equine practitioners (Pinto 2013). Several studies indicate that the sex ratio may be influenced by the method used for synchronization of estrus before insemination (Rorie et al., 1999; Rezagholizadeh et al., 2015; Hall et al., 2022). Women who receive gonadotropins or clomiphene to induce ovulation produce significantly more daughters, supporting the idea that gonadotropins and/or sex steroids can influence the human sex ratio early on (James 1996). It is unclear whether this is due to influences at fertilization or influences on the maternal environment during embryonic development (Navara 2013).

## Conclusion

In conclusion, value biotechniques in the equine species have advanced in the last decade and equine breeders have begun to question the possibilities of interference in determining the sex of foals. The determination of sex is important, as the sex of the foal has a great influence on its commercial value. In our study, we observed that the simple logistic regression model showed that mares and stallions under 15 years old, mares with ovulation time less than 24 hours and treated with Deslorelin had a higher probability of having male foals, but the Pearson's chi-square test showed that foals gender were not influenced by the variables studied.
